# Acute Cerebral Stroke with Multiple Infarctions and COVID-19, France, 2020

**DOI:** 10.3201/eid2609.201791

**Published:** 2020-09

**Authors:** Souheil Zayet, Timothée Klopfenstein, Róbert Kovẚcs, Silviu Stancescu, Beate Hagenkötter

**Affiliations:** Nord Franche-Comté Hospital, Trévenans, France

**Keywords:** Coronavirus diseases, 2019 novel coronavirus disease, COVID-19, SARS-CoV-2, severe acute respiratory syndrome coronavirus 2, respiratory diseases, zoonoses, viruses, pneumonia, central nervous system, stroke, infarction, France

## Abstract

We describe 2 cases in coronavirus disease patients in France involving presumed thrombotic stroke that occurred during ongoing anticoagulation treatment for atrial fibrillation stroke prophylaxis; 1 patient had positive antiphospholipid antibodies. These cases highlight the severe and unique consequences of coronavirus disease–associated stroke.

Coronavirus disease (COVID-19) is an infectious disease caused by severe acute respiratory syndrome coronavirus 2 (SARS-CoV-2). Evidence increasingly shows that SARS-CoV-2 is not always confined to the respiratory tract but can induce neurologic diseases (*1*). Several studies have reported that acute ischemic stroke can develop in COVID-19 patients (*1*–*6*). We describe 2 COVID-19 patients who had multiple cerebral infarctions; 1 patient had positive antiphospholipid antibodies.

On March 25, 2020, an 84-year-old man with a history of diabetes mellitus, arterial hypertension, coronary heart disease, peripheral arterial disease, and atrial fibrillation (treated with apixaban [2.5 mg orally 2×/d]) sought care for respiratory symptoms, including dyspnea and cough. At admission, physical examination revealed a blood pressure of 120/70 mm Hg, irregular heartbeat (100 beats/min), fever (39°C), and bilateral crackling sounds on pulmonary auscultation. Laboratory findings revealed low leukocyte count and lymphopenia (Appendix). Chest radiograph showed a bilateral interstitial infiltrate. Real-time reverse transcription PCR on a nasopharyngeal swab specimen confirmed COVID-19. 

Supportive treatment began (oxygen support, antimicrobial drugs [ceftriaxone 1 g by intravenous (IV) infusion/d], and hydroxychloroquine [200 mg orally 2×/d]), and the same dosage of apixaban was continued. On April 3 (day 9 of hospitalization), dysarthria, left hemiplegia, and alteration of consciousness developed. Brain magnetic resonance imaging revealed acute ischemic stroke in multiple vascular areas (Figure). We switched the anticoagulation medication from apixaban to IV unfractionated heparin [18 UI/kg/h]). On April 6 (day 12), the patients Glasgow coma scale score was 3/15 (eye opening = 1, motor response = 1, verbal response = 1), and severe acute respiratory distress developed. No neurologic recovery occurred, and the patient did not undergo subsequent brain imaging. Mechanical ventilation was not possible (high Charlson comorbidity index), and the patient died on April 12 (day 18).

**Figure:**
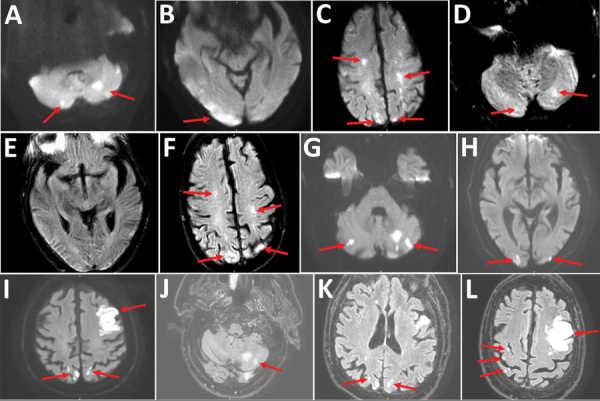
Cerebral magnetic resonance image (MRI) showing acute ischemic stroke in multiple vascular areas of 2 coronavirus disease patients, France. A–F) Patient 1. Diffusion weighted imaging (DWI) showed hyperintensive lesions of bilateral cerebellar hemispheres (arrows, A), right occipital cortex (arrows, B), bilateral centrum semiovale and bilateral parietal cortex (arrows, C). A part of the lesions are already hyperintensive in FLAIR (fluid-attenuated inversion recovery) sequences (arrows, D, F). Normal FLAIR sequence of the right occipital cortex; early stroke MRI (E). MRI quality is reduced because of dental artifact. G–L) Patient 2. Cerebral MRI showed multiple small ischemic infarctions with hyperintensive lesions (arrows) in bilateral cerebellar hemispheres (DWI [G], FLAIR [J; only left hemisphere]), bilateral occipital cortex (DWI [H], FLAIR [K]), main infarction in the left frontal lobe and small biparietal infarctions (DWI [I], FLAIR [L]).

On April 3, a 74-year-old man with a history of multiple cardiovascular diseases, such as atrial fibrillation treated with rivaroxaban (20 mg orally 1×/d), sought care for influenza-like illness and confusion. His work colleagues had noticed disorientation during his activity as a truck driver. At admission, physical examination revealed hypertension (230/70 mm Hg) and irregular heartbeat (86 beats/min). He was febrile (38.3°C) and had crackling sounds on pulmonary auscultation. Neurologic examination showed nonfluent aphasia. COVID-19 was diagnosed from results of real-time reverse transcription PCR, microbiologic testing, and computed tomographic thoracic imaging (Appendix). Brain computed tomographic scan revealed many recent ischemic infarctions in different vascular areas, and magnetic resonance imaging of the brain confirmed this finding (Figure). As with patient 1, this patient had no non–central nervous system thrombotic events (e.g., pulmonary embolisms, abdominal visceral infarction). Treatment began with IV unfractionated heparin (18 UI/kg/h), hydroxychloroquine (200 mg orally 2×/d), and antimicrobial drugs (ceftriaxone 1g by IV infusion/d). The patient’s aphasia regressed, and he was discharged on April 20.

Several factors can cause acute ischemic stroke, but the primary ones are arterial and cardiac embolism, arterial wall disease, and variants of those conditions. Both of these patients had concurrent cardiovascular conditions, particularly atrial fibrillation, although both were adequately treated with anticoagulants. Hematologic derangements, including lymphopenia and leukopenia, are associated with ischemic stroke and are predictors of worse prognosis with stroke (*7*). A systematic review and meta-analysis identified lymphopenia as one of the most prevalent laboratory results described in COVID-19 (35%–72%) (*8*), and we observed it in these 2 patients. Many infectious agents have been implicated as potential causes of cerebral stroke, such as herpes simplex virus, varicella zoster virus, *Treponema pallidum*, *Mycobacterium tuberculosis*, and *Aspergillus* spp.; acute bacterial meningitis has also been implicated (*9*). Multiple brain localizations have previously been described with other viruses that lead to cerebrovascular complications through various mechanisms, including multifocal vasculopathy, focal infiltrative vasculitis and vasospasm, and direct vessel wall invasion and thrombus formation (*10*). 

In this rapidly emerging epidemic, several cases have reported strokes in SARS-CoV-2–infected patients (*1*–*6*). However, the unique feature in the patients we report is multiple simultaneous strokes. These cases involved presumed thrombotic stroke that occurred during ongoing anticoagulation for atrial fibrillation stroke prophylaxis. Given the increasing realization that COVID-19 might be associated with hypercoagulability, the concurrent presence of anticoagulation with direct oral anticoagulants should not be reassuring as preventive. 

Other authors suggest that the presence of antiphospholipid antibodies, such as anticardiolipin antibodies, as well as anti–β_2_-glycoprotein I antibodies might rarely lead to multiple thrombotic cerebral events (*5*). In the patients we report, subsequent serologic testing showed anticardiolipin antibodies (IgM) in patient 1. We did not conduct functional testing (such as dilute Russell viper venom time). However, antiphospholipid antibody syndrome in cases of stroke cannot be diagnosed until positive antibodies persist after multiple months.

The association between cerebral stroke and COVID-19 requires more attention. Coagulability dysfunction and possibly antiphospholipid antibody syndrome may contribute to thromboembolic events in the central nervous system. Further investigation is required to determine the prognostic role of the presence of antiphospholipid antibodies in COVID-19.

Appendix Additional data for 2 coronavirus disease patients with acute cerebral stroke, France 2020.
