# Supplementary material for: Acute Cerebral Stroke with Multiple Infarctions and COVID-19, France, 2020
Source: Emerg Infect Dis. 2020 Sep;26(9):2258–60. doi: 10.3201/eid2609.201791 (PMC7454094; doi:10.3201/eid2609.201791)
Supplement: Appendix — Additional data for 2 coronavirus disease patients with acute cerebral stroke, France 2020. [file 20-1791-Techapp-s1.pdf]

Article DOI: https://doi.org/10.3201/eid2609.201791

Acute Cerebral Stroke with Multiple Infarctions and COVID-19, France, 2020

Appendix

Appendix Table. Clinical, microbiological and radiologic data of the 2 COVID-19 patients with acute cerebral stroke, Nord Franche-Comte Hospital, France, 2020

Characteristic	Patient 1	Patient 2
Age, y	84	74
Sex	M	M
Comorbidities	Diabetes mellitus, AF, coronary heart disease, peripheral arterial disease	Diabetes mellitus, AF, hypertension
Anticoagulant treatment	Apixaban	Rivaroxaban
Days from disease onset to thrombotic event	9	Not determined
Saturation O_2 on admission, %	95	97
Biological data on admission		
Complete blood count (reference)		
White-cell count/mm^3 (4,000–10,000/mm^3)	2,900	11,700
Lymphocytes/mm^3 (1,500–4,000/mm^3)	800	1,200
Platelets count/mm^3 (150,000–450,000/mm^3)	73,000	270,000
Hemoglobin, g/dL (13.5–17.5 g/dL)	12.6	12
Albumin, g/L (35–50 g/L)	16.7	28.6
Alanine aminotransferase, U/L (8–45 U/L)	38	20
Aspartate aminotransferase, U/L (10–40 U/L)	66	22
Lactate dehydrogenase, U/L (190–430 U/L)	685	326
Creatinine, μmol/L (65–120 μmol/L)	102	89
Creatine kinase, U/L (15–130 U/L)	692	24
High-sensitivity cardiac troponin I, pg/mL (<45 pg/mL)	46.6	1,536
Prothrombin time, s (11–12.5 s)	11.8	12.3
Fibrinogen, g/L (2–4 g/L)	6.6	5.5
D-dimer, mg/L (<500 mg/L)	19,181	1,376
Triglycerides, g/L (<1.5 g/L)	1.4	1.5
Serum ferritin, μg/L (18–270 μg/L)	1,572	309
C-reactive protein, mg/L (<5 mg/L)	109	30
Antiphospholipid antibodies (IgM and IgG Anticardiolipin, IgM and IgG anti-β_2-glycoprotein)	Anticardiolipin IgM positive	All negative
Radiologic data		
Thoracic imaging features	Bilateral pulmonary infiltrates	Bilateral subpleural ground-glass opacities with a basal distribution
Brain MRI with vascular sequences (2D-TOF, 3D-TOF, and 3D T1 sequence with gadolinium enhancement)	Multiple cerebral infractions in bilateral cerebellar hemispheres, bilateral centrum semiovale, bilateral parietal lobe and right occipital lobe	Multiples cerebral infractions in bilateral cerebellar hemispheres, bilateral parietal occipital lobe and left frontal lobe
RT-PCR SARS-CoV-2RNA		
Respiratory specimen	Nasopharyngeal swab	Nasopharyngeal swab and sputum sample
Result	Positive	Positive
Viral load in respiratory specimen, log copies/mL (normal mean: 5.5 log copies/mL)†	6.8	4.3

*AF, atrial fibrillation; COVID-19, coronavirus disease; MRI, magnetic resonance imaging; RT-PCR, real-time reverse transcription PCR.
†In Nord Franche-Comté Hospital during March 1–14, 2020, COVID-19 was diagnosed in 68 patients without any neurologic symptoms. Patients with acute cerebral stroke each had measured nasopharyngeal viral loads (5.6 log copies/mL) similar to the mean viral load in COVID-19 patients without stroke (5.5 log copies/mL) in this facility.
